# Gonorrhœa

**Published:** 1863-08

**Authors:** M. H. Collis


Gonorrhœa—*Injections.*—The main treatment of gonorrhœa should be by injections, copavia and cubebs being seldom necessary. The rule for their use is simple: If the inflammation be severe, let the solution be weak and frequently used; if it be of chronic type, let the solution be strong and seldom used. This rule is not only applicable to the treatment of gonorrhœa, but it may be extended (with necessary modification to treatment of all inflammations, and to the use of internal remedies, as well as of external applications. There is a general law regulating the actions of a large class of remedial bodies. The terms of it would run something in this form: "The more acute the disease, the more frequent and the weaker the remedy; the more chronic, the stronger and less frequently applied." One of the most reliable astringents for the cure of gonorrhœa is alum. In the most acute form of gonorrhœa, when the discharge is profuse, thick, and glutinous—the lips of the urethra red, villous, and pouting—the patient should be directed to pour a small jug of cold water over the organ, and immediately inject a syringeful of solution of alum of the strength of half a grain to the ounce. This injection should be repeated every half hour for the first day, and as often at night as the intervals of sleep will allow. Along with this a saline purgative, and tartar emetic in minute doses, should be given. Next day the injections may be doubled in strength, and used every hour. Probably in forty-eight hours more the discharge will have ceased entirely, the injections must, however, be continued a week or so, of the strength of half a drachm to the eight ounces, three times a day; otherwise a relapse may occur which will be harder to cure than the original clap.—(Dr. M. H. Collis.)—*Id.*

